# A Two-Stage Approach Integrating Provisional Biomaterial-Mediated Stabilization Followed by a Definitive Treatment for Managing Volumetric Muscle Loss Injuries

**DOI:** 10.3390/jfb15060160

**Published:** 2024-06-06

**Authors:** Andrew R. Clark, Jonathan Kulwatno, Sergey S. Kanovka, George J. Klarmann, Claudia E. Hernandez, Roman M. Natoli, Todd O. McKinley, Benjamin K. Potter, Christopher L. Dearth, Stephen M. Goldman

**Affiliations:** 1 Extremity Trauma and Amputation Center of Excellence, Defense Health Agency, Falls Church, VA 22042, USA; 2 Department of Surgery, Uniformed Services University of the Health Sciences, Bethesda, MD 20814, USA; 3 The Henry M. Jackson Foundation for the Advancement of Military Medicine, Bethesda, MD 20817, USA; 4 4D Bio3 Center for Biotechnology and Department of Radiology and Radiological Sciences, Uniformed Services University of the Health Sciences, Bethesda, MD 20817, USA; 5 The Geneva Foundation, Tacoma, WA 98402, USA; 6 C2 Alaska, LLC, San Antonio, TX 78249, USA; 7 Department of Orthopaedic Surgery, Indiana University School of Medicine, Indianapolis, IN 46202, USA; 8 Department of Orthopaedic Surgery, Walter Reed National Military Medical Center, Bethesda, MD 20889, USA

**Keywords:** skeletal muscle, trauma, orthopedics, military medicine, polymers

## Abstract

Treatment of volumetric muscle loss (VML) faces challenges due to its unique pathobiology and lower priority in severe musculoskeletal injury management. Consequently, a need exists for multi-stage VML treatment strategies to accommodate delayed interventions owing to comorbidity management or prolonged casualty care in combat settings. To this end, polyvinyl alcohol (PVA) was used at concentrations of 5%, 7.5%, and 10% to generate provisional muscle void fillers (MVFs) of varying stiffness values (1.125 kPa, 3.700 kPa, and 7.699 kPa) to stabilize VML injuries as part of a two-stage approach. These were implanted into a rat model for a duration of 4 weeks, then explanted and either left untreated (control) or treated through minced muscle grafting (MMG). Additional benchmarks included acute MMG and unrepaired groups. At the MVF explant, the 7.5% PVA group exhibited superior neuromuscular function compared to the 5% and 10% PVA groups, the least fibrosis, and the largest median myofiber size among all groups at the 12-week endpoint. Despite the 7.5% PVA’s superiority amongst the two-stage treatment groups, neuromuscular function was neither improved nor impaired relative to acute treatment benchmarks. This suggests that the future success of a two-stage VML treatment strategy will necessitate a more effective definitive intervention.

## 1. Introduction

Multi-staged treatment strategies, exemplified by techniques like the induced membrane method, have found application in the treatment of traumatic (extremity) injuries, notably focusing on the restoration of long bone fractures [1,2]. These procedures, however, frequently neglect the significance of acutely addressing soft tissue injuries, even though the types of injuries requiring multi-staged treatment plans are typically associated with substantial damage to surrounding soft tissues, such as Gustilo–Anderson type III fractures [3]. Despite this clinical unmet need, there is a significant lack of research dedicated to investigating multi-staged treatment approaches specifically designed to acutely address soft tissue injuries, which could serve as valuable additions to the current multi-staged treatments for skeletal injuries. Presently, it is unclear whether similar multi-staged treatment strategies would have adverse effects or provide similar benefits for severe muscle injuries, i.e., volumetric muscle loss (VML). To that end, this study aims to investigate the effectiveness of a two-stage treatment approach that parallels the induced membrane technique but is adapted for VML. In this approach, a provisional muscle void filler (MVF) is implanted within the VML defect for a period of time, subsequently removed, and replaced with a definitive treatment. This is in contrast to the majority of research that has been conducted to develop treatments for VML, as they have focused on delivering definitive treatments as early as possible [4,5,6,7]. Any beneficial effects of these early treatments may be limited or negated by the putative high rates and risk of early infection following severe injury, the inability to perform an early range of motion of adjacent joints, the need for subsequent additional procedures for other complicating or related injuries, and so forth. Furthermore, the delayed application of virtually any treatment of VML without the use of MVFs may be further complicated by the formation of scar tissue and/or soft tissue complications (i.e., the loss of potential space fur to the former).

When VML is left untreated due to the absence of, or delay in, a definitive treatment, the innate pathobiology results in the deposition of fibrotic tissue and resultant fibrosis, contracture, limitations in range of motion, and impaired function [8]. A two-staged treatment strategy could be beneficial for VML by providing an acute provisional treatment to subside the acute sequelae of injury (e.g., inflammation, infection, etc.) before providing a definitive treatment, including those that attempt to initiate the regeneration process. There are also practical reasons that a multi-staged treatment, even if it does not facilitate increased treatment efficacy, may be clinically necessary. For example, while ostensibly successful treatments are currently being developed for VML, research has demonstrated that a cellular component is likely necessary for meaningful recovery [9]. Autologous cell-based therapies often require extensive preparation, involving the acquisition, expansion, and culturing of the patient’s own cells. These processes contribute to the considerable latency between the time of injury and the delivery of treatment to the patient [10,11]. Additionally, a growing concern for the U.S. Department of Defense is the need to provide prolonged care for injured soldiers and civilians in a far-forward setting with limited access to medical supplies [12]. Thus, there is an unmet need for the development of an implantable MVF that can stabilize the VML injury and prevent the intrinsic pathological response as a means to enable the success of a delayed definitive treatment.

Our previous research investigated different biomaterials for their use as a MVF [13], the results of which established polyvinyl alcohol (PVA) as the lead candidate. PVA has several notable attributes as it relates to its ability to serve as a MVF: nondegradable, elicits a relatively low immune and foreign body response [14], and its simplicity and stability, which allow for potential use in low-resource settings such as a prolonged care scenario [15]. Furthermore, by utilizing PVA with methacrylate functional groups, we were able to initiate its gelation in situ utilizing ultraviolet light, thereby allowing the PVA to conform to the unique geometry of the VML defect volume. In order to optimize its effectiveness, we evaluated a range of PVA concentrations with resulting differences in stiffness, as the physical properties of the implanted material could possibly affect its efficacy as a MVF and have varying effects on the surrounding tissue.

While more advanced definitive therapies for VML are still in development [16], we utilized minced muscle grafting (MMG) as a model therapy herein. MMG has been readily studied in preclinical models of VML for its ability to partially restore muscle tissue within the defect and facilitate associated functional improvements [17,18,19]. In essence, MMG is a cell transplant containing the cells likely needed to regrow muscle (e.g., satellite cells, endothelial cells, fibroblasts, resident macrophages, etc.). While a MMG structure does not have the 3D structure of a sophisticated tissue-engineered construct, it has immense regenerative potential, as evidenced by its ability to form contractile regenerates in vivo in the absence of an existing muscle [20].

The goal of this study was to evaluate a multi-staged treatment strategy in which a VML injury is acutely stabilized through a PVA MVF. In the second stage, the MVF is removed and replaced with the definitive MMG treatment that aims to regenerate the lost tissue within the VML defect. Such a multi-staged treatment strategy would enable a delayed definitive treatment approach if required due to clinical care considerations and/or practical limitations, such as prolonged forward care or the time required to create a cell-based treatment. Our hypothesis was that a PVA MVF can effectively stabilize a VML defect for a period of 28 days and then be replaced with a definitive MMG treatment without a decrease in MMG therapeutic efficacy. The success of such a multi-staged approach would provide clinicians, especially those in far-forward resource-scarce locations, with a valuable tool for treating traumatic injuries and be potentially used alongside other multi-staged treatment strategies, such as the induced membrane technique for the treatment of segmental bone defects.

## 2. Materials and Methods

### 2.1. Biomaterial Preperation

The PVA solutions utilized in this study were of acrylamide-modified PVA (at the concentrations of 5%, 7.5%, or 10% in water) with a molecular weight of 70 kDa (BioCure, Atlanta, GA, USA). Additionally, 1% *v/v* of Irgacure 2959 at a concentration of 1000 mg/mL in DMSO (Advanced Biomatrix, Carlsbad, CA, USA) was incorporated as the photoinitiator. The resulting PVA solutions were polymerized via ultraviolet (UV) light in both in vitro and in vivo experiments.

### 2.2. Biomaterial Rheology

The rheological properties of PVA biomaterials were assessed using an MCR302 rheometer (Anton Parr, Graz, Austria), equipped with a parallel plate (PP25), and controlled by RheoCompass software v1.24. Prior to initiating polymerization, PVA solutions were allowed to reach a temperature of 37 °C, after which ultraviolet (UV) light was employed for initiation. During the gelation process, the rheometer applied a 2% shear strain (within the linear viscoelastic range for PVA solutions) at a frequency of 1 Hz, maintaining a 0.7 mm gap with normal force control enabled to accommodate expansion or contraction.

### 2.3. Surgical Procedures

All animal procedures were approved by the Institutional Animal Care and Use Committee at the Uniformed Services University of the Health Sciences (Protocol# SUR-23-014) and were conducted in AAALAC-accredited facilities of the Department of Laboratory Animal Research. All animals were exposed to a 12 h light/dark cycle and had ad libitum access to food and water. All experiments utilized adult male Lewis rats starting at 10–12 weeks old and ~350 g (Charles River Laboratories) and were block randomized to experimental groups. Animals received unilateral VML injuries in the tibialis anterior (TA) muscle using previously established methods [21]. Prior to surgery, animals received a subcutaneous dose of Ethiqa XR (buprenorphine-extended release; 0.65 mg kg^−1^ bodyweight) for analgesia. Animals were anesthetized using isoflurane (5% initially, 1–3% for maintenance). A lateral incision was made through the skin and fascia, and the TA muscle was exposed. A 6 mm full-thickness biopsy was removed from the middle third of the muscle belly to create the VML injury. Any bleeding was stopped by applying pressure. Once bleeding stopped, rats received one of the following: (1) ~100 µL of the MVF precursor (10% PVA, 7.5% PVA, or 5% PVA) was pipetted in and immediately exposed to UV light for 30 s; (2) MMG made by mincing the biopsied muscle and inserting it back into the defect (acute MMG); or (3) nothing was added to the defect (no repair). The wound was then closed. Rats that received a MVF underwent a second procedure after 4 weeks. During this procedure, the TA was exposed similar to the VML surgery, the MVF was removed and replaced with either (1) a ~90 mg MMG from a donor rat or (2) nothing added to the defect. To assess MVF implantation, some animals that received a MVF were euthanized at 1 day or 4 weeks post-implantation. The multi-staged treatment groups were euthanized 8 weeks after the second procedure. Animals that received an acute MMG or no repair during the VML procedure were euthanized 12 weeks following the VML surgery. Animals were euthanized via intracardiac delivery of Euthasol (pentobarbital sodium and phenytoin sodium) while under anesthesia. The TA and extensor digitorum longus (EDL) muscles were harvested post-mortem, separately weighted, and snap frozen in an optimal cutting temperature compound via liquid nitrogen-cooled isopentane for histological analyses. A total of 100 rats were used in these experiments.

### 2.4. Neuromuscular Functional Assessment

The animals were placed under anesthetic restraint using isoflurane at a concentration of 1–3% and provided with supplemental heating at 37 °C throughout the procedure. To assess neuromuscular function, we employed a dual-mode muscle lever system (Model 305C, Aurora Scientific, Inc). The foot was securely fastened to a foot pedal connected to a force transducer, with the ankle positioned at a 90° angle. Torque was calculated by the resulting force measurements multiplied by the length of the foot pedal. Percutaneous needle electrodes were utilized to stimulate the common peroneal nerve. The voltage required to induce maximum tetanic contractions (150 Hz, 0.1 ms pulse width, and a 400 ms train) was determined for each rat and used for the remainder of the procedure. For the endpoint assessment, the extensor digitorum longus tendon was cut, and stimulations were subsequently applied at a range of frequencies, spanning from 10 to 200 Hz. This assessment was performed one day after MVF removal (4 weeks post-VML) and at the endpoint of 12 weeks post-VML.

### 2.5. Histology

Frozen samples were cross-sectioned at a thickness of 7 μm in the middle of the VML defect and fixed with formalin for downstream processing. Picrosirius red (PSR) staining, which stains collagen red and cells yellow, was performed with a PSR kit (Abcam, ab150681) following the manufacturer’s protocol. Slides were sealed with Micromount^®^ Mounting Medium (Leica, 3801730). PSR cross-sections were used to calculate rectangularity (the ratio of the area of biomaterial to the area of minimum bounding rectangularity) [22]. Collagen and tissue area quantification were performed through ImageJ using the k-means clustering macro. We stained the sarcolemma by incubating slides in wheat germ agglutinin (WGA) with Alexa Fluor™ 488 conjugate (1:500 Invitrogen, W11261) for one hour. The slides were then washed and mounted with VECTASHIELD^®^ Vibrance™ Antifade Mounting Medium with DAPI (Vector, H1800). WGA fiber count and size were performed using the Myosoft 14 macro in ImageJ. All slides were imaged at 10× magnification with an Axio Scan Z1 (Zeiss; Oberkochen, Germany).

### 2.6. Statistical Analysis

Unless stated otherwise, all data are represented as means with standard deviations (SDs). The results were statistically analyzed by GraphPad Prism 10 software. Data comparing multiple factors were analyzed through a 1-, 2-, or 3-way ANOVA, including interaction effects. Statistically significant ANOVAs then underwent a Holm–Šídák post-hoc test. Statistical tests for the compression testing were run on log-transformed data in order to make the data more homoscedastic. Statistical significance for tests were determined by *p*-values less than 0.05.

## 3. Results

**Figure 1:**
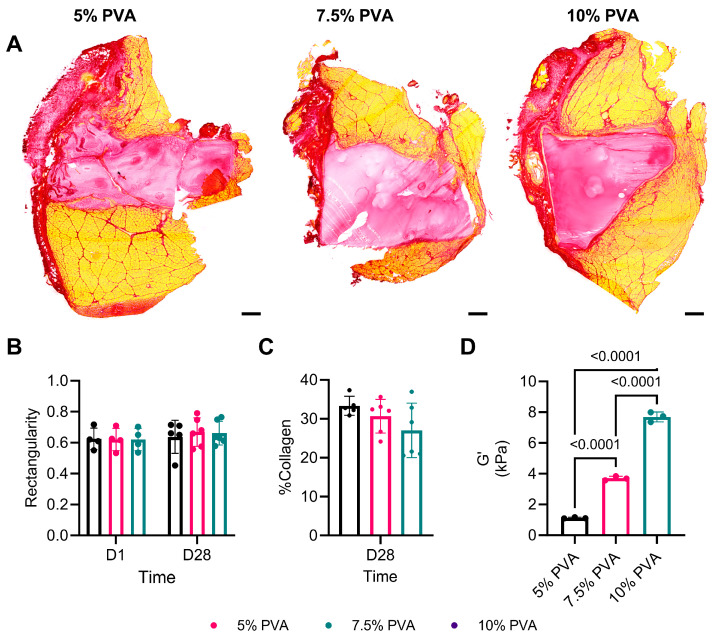
(**A**) Representative images of picrosirius red-stained cross-sections of volumetric muscle loss (VML)-injured tibialis anterior muscles 28 days after muscle void filler (MVF) implantation. (**B**) Rectangularity measurements of MVFs from cross-sections of the middle of the VML injury. (**C**) Quantification of the percentage of the cross-sectional area that stained positive for collagen. (**D**) Storage modulus of MVFs made from each concentration of polyvinyl alcohol (PVA). All data were analyzed first via a 1- or 2-way ANOVA, followed by the Holm–Šídák post-hoc test. Post-hoc *p*-values less than 0.05 are listed for all comparisons. Data are represented as the mean ± SD.

Three concentrations of PVA (5%, 7.5%, and 10%) were polymerized to form hydrogels by crosslinking with UV light exposure. In vitro rheological testing measured storage moduli of 1.125 ± 0.044 kPa (5% PVA), 3.700 ± 0.136 kPa (7.5% PVA), and 7.699 ± 0.321 kPa (10% PVA) (Figure 1D). Upon in situ polymerization within a VML defect, the PVA solutions successfully created a MVF that conformed to the defect’s volume and preserved the space for a duration of 28 days (Figure 1A). Quantification of the rectangularity of the MVFs showed no changes between 1 and 28 days post-implantation or with the stiffer PVA concentrations (5% PVA: 0.62 ± 0.07 (D1) and 0.64 ± 0.11 (D28); 7.5% PVA: 0.62 ± 0.07 (D1) and 0.67 ± 0.09 (D28); 10% PVA: 0.62 ± 0.07 (D1) and 0.66± 0.08 (D28)) (Figure 1B). There were also no differences in collagen content between any of the groups 28 days post-MVF implantation (*p* = 0.1519) (Figure 1C).

**Figure 2:**
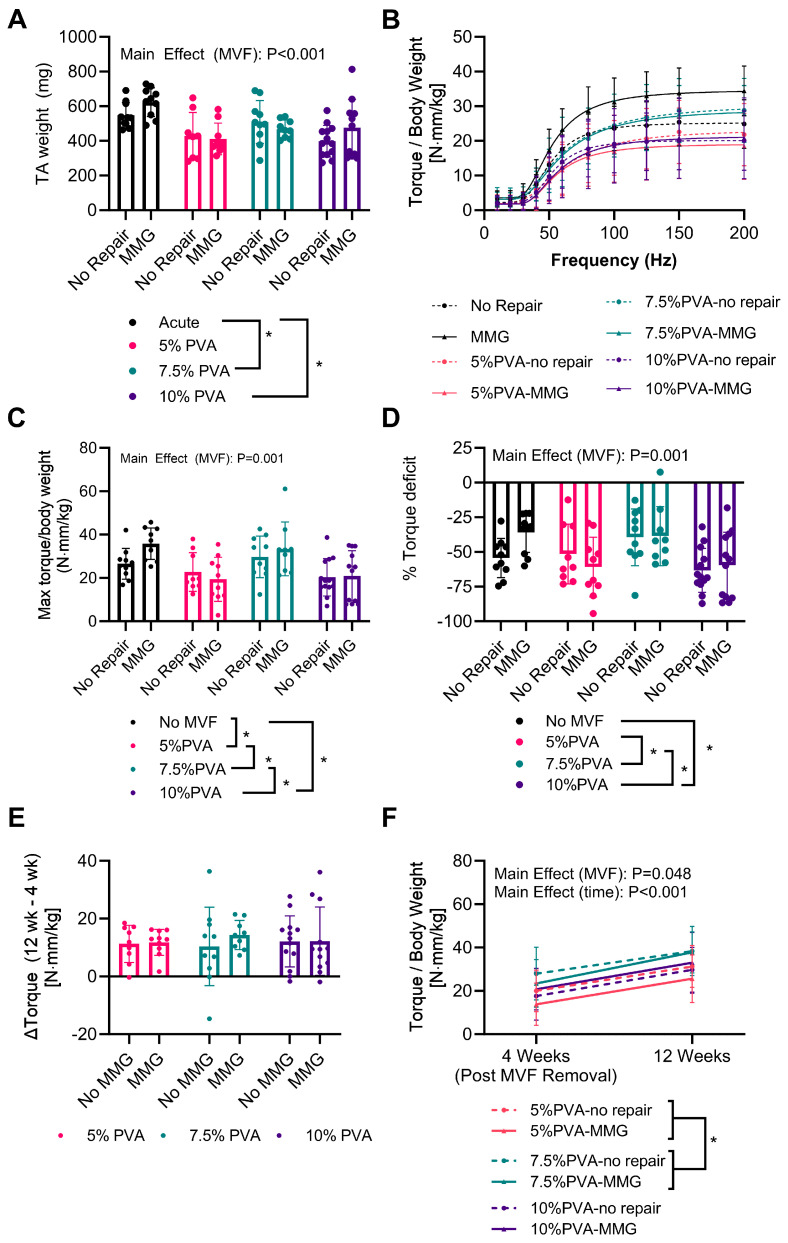
(**A**) Weight of tibialis anterior (TA) muscles 12 weeks after VML. (**B**) Torque production of dorsiflexion (after extensor digitorium longus (EDL) tenotomy) at peroneal nerve stimulation frequencies of 10–200 Hz with the best fit sigmoidal fit line. (**C**) Maximal torque production of dorsiflexion (after EDL tenotomy) with supramaximal-stimulated peroneal nerve. (**D**) Torque deficit of injured limbs expressed as a percentage of uninjured contralateral. (**E**) Longitudinal measurements of maximal torque production of dorsiflexion with supramaximal-stimulated peroneal nerve 1 day after MVF removal and 8 weeks after MVF removal. (**F**) Change in maximal dorsiflexion torque after MVF removal and 8 weeks after MVF removal. All data were analyzed first via a 2- or 3-way ANOVA, followed by a Holm–Šídák post-hoc test comparing differing groups with significant main effects. * Indicates *p* < 0.05 from the post-hoc tests. Data are represented as the mean ± SD.

We next utilized the MVFs as a provisional treatment and subsequently removed them prior to MMG implantation as a definitive treatment. MMG had no effect on TA weights over the 8-week period (main effect *p* = 0.28); however, the acute treatment had the greatest muscle weights compared to the multi-staged treated groups (acute vs. 5% PVA: +168.3 mg, *p* = 0.0005; vs. 7.5% PVA: +96.7 mg, *p* = 0.0642; vs. 10% PVA: +148.3 mg, *p* = 0.0008) (Figure 2A). The neuromuscular strength of dorsiflexion was measured after 12 weeks post-VML injury, with the distal tendon of the EDL removed to isolate the force production caused by the TA. MMG implantation was not found to have an impact on neuromuscular strength (main effect *p* = 0.233; interaction (MMG × MVF) *p* = 0.218). Rats that underwent multi-staged treatment with the 5% PVA MVF and 10% PVA MVF had lower maximal torque production (normalized to body weight) than either the acute-treated animals (acute vs. 5% PVA: +10.11 N·mm/kg, *p* = 0.008; acute vs. 10% PVA: +10.55 N·mm/kg, *p* = 0.0023) or the multi-staged-treated rats with the 7.5% PVA MVF (7.5% PVA vs. 5% PVA: +10.51 N·mm/kg, *p* = 0.0064; 7.5% PVA vs. 10% PVA: +10.94 N·mm/kg, *p* = 0.0020). The 7.5% PVA multi-staged-treated rats demonstrated similar maximal torque production to the acutely treated rats (acute vs. 7.5% PVA: −0.40 N·mm/kg, *p* = 0.9988) (Figure 2C). These findings were mirrored in the torque production at higher frequencies, but they converged at submaximal stimulation frequencies (interaction *p* < 0.0001) (Figure 2B). However, when the torque is normalized to the contralateral limb (expressed as a percentage deficit), the acute treatment groups were only different from the 10% PVA multi-staged treatment group (acute vs. 5% PVA: +10.99%, *p* = 0.297; vs. 7.5% PVA: −6.178%, *p*= 0.754; vs. 10% PVA: +16.26, *p* = 0.033). Meanwhile, the 7.5% PVA multi-staged treatment group had the highest amount of functional recovery of the multi-staged-treated groups (7.5% PVA vs. 5% PVA: +17.17%, *p* = 0.039; vs. 10% PVA: +22.44%, *p* = 0.002). MMG had no apparent effect (main effect: *p* = 0.1730, interaction (MMG × MVF) *p* = 0.4372) (Figure 2D).

Further non-terminal in vivo functional assessments were conducted without cutting the EDL immediately after MVF removal surgery and 8 weeks later at the endpoint (refer to Figure 2E,F). Functional recovery following MVF removal was consistent across all groups, with a global average of 12.00 ± 8.93 N·mm/kg over these 8 weeks (MVF: *p* = 0.959; MMG: *p* = 0.525; interaction (MVF x MMG): *p* = 0.7678). Notably, the 7.5% PVA-treated group exhibited increased function at the time of MVF removal (7.5% PVA vs. 5% PVA: +8.908 N·mm/kg, *p* = 0.032; vs. 10% PVA: +6.69 N·mm/kg, *p* = 0.0844), and this improvement was persistent through the end of the experiment (7.5% PVA vs. 5% PVA: +9.412 N·mm/kg, *p* = 0.0214; 7.5% PVA vs. 10% PVA: +6.78 N·mm/kg, *p* = 0.079).

**Figure 3:**
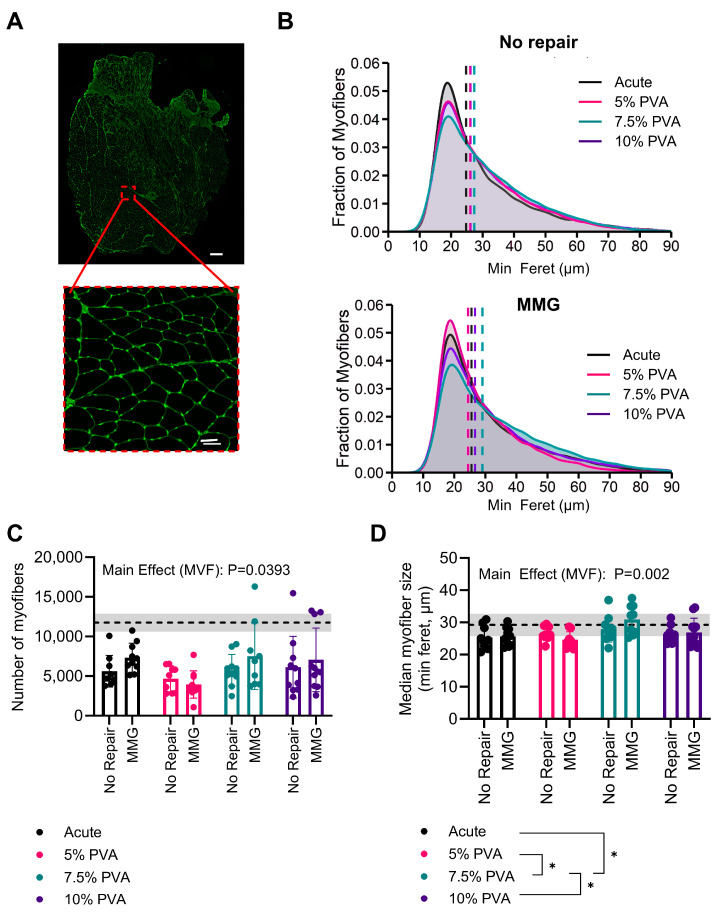
(**A**) Representative image of WGA staining of a VML-injured TA (large image scale bar = 500 μm; magnified image scale bar = 50 μm). (**B**) Kernel density plot of the myofiber size distribution of acute and multi-staged treated groups at experimental endpoint (median denoted by the dashed line). (**C**) Total count of myofibers. Mean ± SD of healthy contralateral limbs reflected in dashed line with shading. (**D**) Median size of myofibers. Mean ± SD of healthy contralateral limbs reflected in dashed line with shading. Data were analyzed first via a 2-way ANOVA, followed by the Holm–Šídák post hoc test. * Indicates *p* < 0.05 from the post-hoc tests. Data in the bar graphs are represented as the mean ± SD. The dashed line and gray shading represent the mean ± SD values from uninjured TA muscles.

Muscle fiber analyses were performed on histological cross-sections of the muscles stained with WGA (Figure 3A,B). There was an effect of MVF concentration on the total number of myofibers (*p* = 0.0393) (Figure 3C). While there were no intergroup differences, the largest difference was between the 5% PVA and the 7.5% PVA groups (−2337 myofibers, *p* = 0.0874). Similarly, there is no effect of MMG on median myofiber size (*p* = 0.510). However, the 7.5% PVA multi-staged treatment groups had the largest median myofibers (vs. acute: +3.977 µm, *p* = 0.0043; vs. 5% PVA: +3.860 µm, *p* = 0.0057; vs. 10% PVA: +2.888 µm, *p* = 0.0408) (Figure 3D).

**Figure 4:**
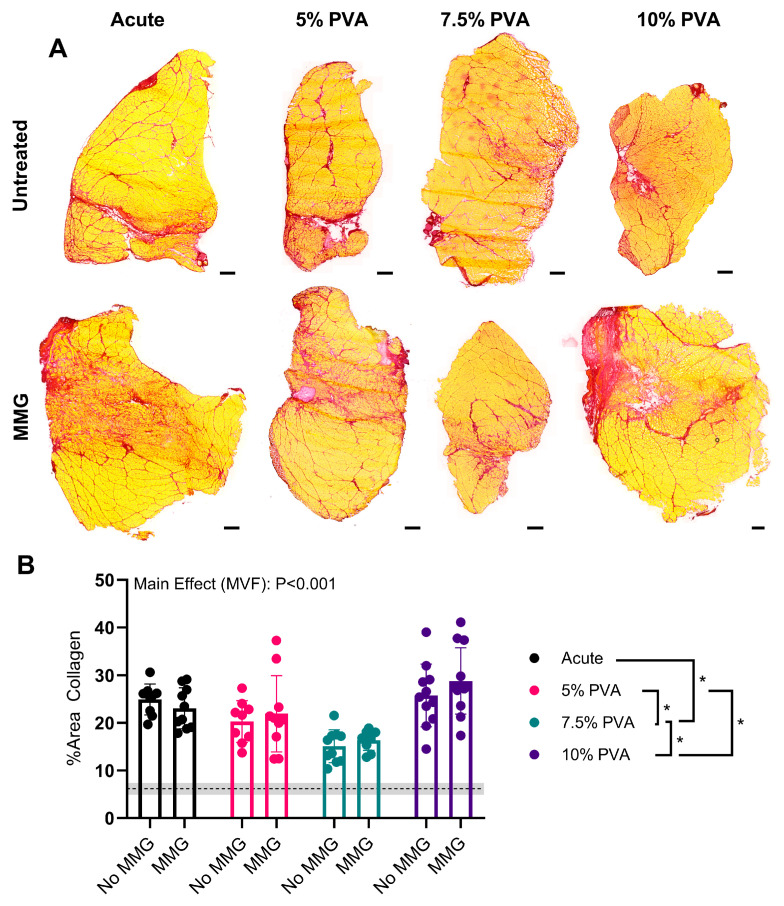
(**A**) Representative images of picrosirius red-staining TA at the experimental endpoint (scale bar = 500 μm). (**B**) Quantification of the percentage of cross-sectional area stained positive for collagen. All data were analyzed first via a 2-way ANOVA, followed by the Holm–Šídák post-hoc test. * Indicates *p* < 0.05 from the post-hoc tests. Data are represented as the mean ± SD. The dashed line and gray shading represent the mean ± SD values from uninjured TA muscles.

PSR-stained harvested cross-sections to assess fibrosis (Figure 4A) demonstrated an increase in collagen area in each treatment group compared to healthy controls (Figure 4B). MMG had no impact on collagen content (main effect, *p* = 0.239); however, the 7.5% PVA multi-staged-treated rats had the lowest amounts of collagen (vs. acute: −8.291%, *p* < 0.0001; vs. 5% PVA: −5.389%, *p* = 0.0175; vs. 10% PVA: −11.55%, *p* < 0.0001). Additionally, the 5% PVA multi-staged-treated rats had lower amounts of collagen than the rats treated with 10% PVA (−6.165%, *p* = 0.0026).

## 4. Discussion

This study aimed to test a two-stage treatment approach for VML, in which a provisional MVF preserves the VML defect and inhibits the progression of the intrinsic pathobiology, followed by a subsequent definitive treatment with MMG in an attempt to regenerate lost muscle tissue within the defect, thereby facilitating restoration of function. This approach is novel, as most experimental interventions for VML administer their treatment immediately after injury in an attempt to initiate regeneration as early as possible [4,5,6,7]. While initiating regeneration as early as possible is attractive in principle, there are many practical factors that may suggest that postponing definitive treatment of VML would be advantageous or even obligatory. Some of these factors are related to the severity and environment of injuries that create a VML (e.g., a vehicle accident or improvised explosive device), which demand priority treatment (e.g., hemorrhages, infections, and fractures) or involve treatment in resource-poor far-forward facilities. In addition, a delay in the definitive treatment of a VML injury may be imposed by the preparation of the therapy, such as a tissue-engineered cell-based construct, which could take weeks or months to prepare. Thus, a clinical unmet need exists for a two-staged treatment strategy to facilitate a delayed definitive treatment.

We utilized three different concentrations of PVA as a MVF over a four-week period. While these concentrations resulted in a range of MVF stiffnesses, they were all equivalent in their ability to maintain the defect volume and the level of collagen deposition from the innate response within the muscle. However, the 7.5% PVA MVF facilitated the highest functional outcome following the 28-day MVF implantation period. Following MVF removal, all multi-staged treatment groups had equivalent functional recovery rates over the proceeding 8 weeks. MMG implantation conferred no functional benefits in any of the two-stage treatment groups. This was surprising due to the wealth of literature showing functional improvements in VML utilizing MMG [17,18,19]. However, this finding is similar to those of one study that attempted to utilize a tissue-engineered muscle in a chronic VML, in which the investigators observed reduced neuromuscular function and the number of myofibers compared to transplantation in an acute VML [23]. A major difference in MMG usage in this study compared to most previous studies was implanting it in a chronic, rather than acute, VML. In a chronic VML injury, the acute injury response has subsided, and the prolonged inflammatory response has already been established or, depending on timing, resolved. It is possible that the balance of cytokines in an acute VML injury is more favorable to the survival and engraftment of an MMG compared to a chronic VML. Additionally, it is possible that MMG donates myogenic stem cells to the surviving musculature, and in a chronic injury, the time window has been missed for most of the myonuclear accretion in the surviving musculature. Another plausible reason for the ineffectiveness of MMG in our study is the thin layer of extracellular matrix (ECM) that formed around the MVF could have acted as a barrier between the MMG and the surviving musculature, preventing its integration. However, in the histology of most samples, this ECM barrier was not evident and was likely remodeled by the completion of this study. While MMG was not an effective regenerative therapy in this study, it is possible for other regenerative therapies and tissue-engineered constructs to be efficacious in this two-staged treatment paradigm [24].

By the completion of this study, the 7.5% PVA multi-staged treatment group had the highest functional recovery of the multi-staged treatment groups. This revealed that the effects of PVA concentration acted in a Goldilocks manner. These functional differences were reflected in 7.5% PVA multi-staged treatment groups having larger muscle fibers and less fibrosis, likely contributing to its increased muscular strength. We do not know the exact mechanisms that are influencing this functional recovery, but we hypothesize that the 7.5% PVA level represents an ideal stiffness where softer or stiffer MVFs alter the programing of myofibroblasts to be more fibrotic and/or cause atrophy of myofibers. This notion is supported by another recent study by Basurto et al. that showed hyaluronic acid-based hydrogel of medium (3.0 kPa stiffness) produced maximum isometric function most similar to healthy muscle when compared to low (~1 kPa) and high (~10 kPa) stiffness hydrogels of the same material using a rat model of a VML affecting the latissimus dorsi muscle [25]. However, the 7.5% PVA two-staged treatment groups showed no functional differences to unrepaired VML. While this demonstrates the importance of a regenerative therapy that can be effective in a chronic VML, it shows promise for the future development of sophisticated MVFs. The current MVF shows proof-of-principle that an appropriate MVF does not worsen a VML. Thus, future MVFs may be able to contain bioactive payloads (e.g., anti-inflammatories and IGF1) that confer a benefit [24,26]. Additionally, the MVF is capable of maintaining a defect volume that could minimize the debridement, soft tissue dissection, or even the advanced soft tissue coverage needed before a delayed regenerative treatment.

The current study has several limitations. The first was that only a single specific VML injury model was used, whereas it can be clinically manifested in many different forms. Our model was a full-thickness injury with remaining continuous muscles fibers both medial and lateral to the zone of injury. The forces applied to a MVF, either through muscle contraction or closing contracture, is likely to be different for different types of injuries (e.g., complete loss of just superficial fibers, only a thin band of remaining continuous fibers medially), muscle pennation angles, and muscle sizes. Thus, while an optimal MVF stiffness was found for this model, it may need further optimization for differing clinical applications. Another limitation was the inability to test the effects of debridement. Due to the small size of the rat TA, with only millimeters of continuous muscle medially and laterally, even the smallest amount of debridement could have a large impact on function. However, the collagen border that forms around the MVF is so thin (<50 µm) that it may not pose a great problem if left undisturbed. However, reports have shown that acute inflammation can be useful for cell transplants [27,28,29], and thus debridement could possibly refresh the wound to an acute injury state that is perhaps more receptive to regenerative therapies. A larger animal model may be more conducive towards testing these experimental questions. Another limitation is that only MMG was used as a definitive treatment. While MMG did not show efficacy in these multi-staged treatment studies, it is unknown whether such a first-stage MVF stabilization process can facilitate other definitive treatments for VML.

## 5. Conclusions

This study employed a two-stage treatment strategy for addressing VML. In this approach, an initial provisional PVA-based MVF was utilized to stabilize the VML wound for a duration of four weeks. Subsequently, the provisional MVF was removed, and definitive treatment was administered through MMG. The investigation revealed a noteworthy “Goldilocks effect” related to the optimal stiffness of the MVF concerning neuromuscular function and fibrosis. When the optimal stiffness of the MVF was applied, the multi-staged approach demonstrated neither a detrimental nor a beneficial impact on neuromuscular function or myofiber count. However, there was a noticeable decrease in fibrosis, along with an increase in median myofiber size. Notably, the utilization of MMG did not appear to exert any influence when incorporated into the multi-staged treatment. This study suggests that further research into alternative delayed regenerative treatments within the context of a multi-staged treatment for VML is warranted.

## Data Availability

The data will be made available upon request.
